# Supplementary material for: Fully stretchable active-matrix organic light-emitting electrochemical cell array
Source: Nat Commun. 2020 Jul 3;11:3362. doi: 10.1038/s41467-020-17084-w (PMC7335157; doi:10.1038/s41467-020-17084-w)
Supplement: Supplementary file 1 — Supplementary Information [file 41467_2020_17084_MOESM1_ESM.pdf]

Fully stretchable active-matrix organic light-emitting electrochemical cell array

Jia Liu[1†], Jiechen Wang[2†], Zhitao Zhang[1†], Francisco Molina-Lopez[1], Ging-Ji Nathan Wang[1], Bob C. Schroeder[1], Xuzhou Yan[1], Yitian Zeng[2], Oliver Zhao[2], Helen Tran[1], Ting Lei[1], Yang Lu[3], Yi-Xuan Wang[1,4], Jeffrey B.-H. Tok[1] Reinhold Dauskardt[2], Jong Won Chung[5], Youngjun Yun[5]* and Zhenan Bao[1]*

[1]Department of Chemical Engineering, Stanford University, CA 94305, USA

[2]Department of Materials Science and Engineering, Stanford University, CA 94305, USA

[3]Beijing National Laboratory for Molecular Sciences (BNLMS), College of Chemistry and Molecular Engineering, Peking University, Beijing 100871, China

[4]Tianjin Key Laboratory of Molecular Optoelectronic Sciences, Department of Chemistry, School of Science, Tianjin University, Tianjin 300072, China

[5]Samsung Advanced Institute of Technology

*These authors contributed equally to this work.

†Corresponding authors. Email: zbao@stanford.edu, youngjun.yun@gmail.com

Supplementary Figures and Figure Legends

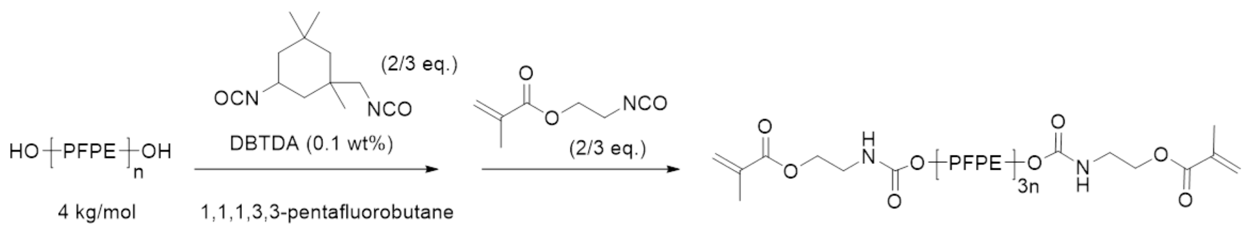

Supplementary Figure 1. Synthesis of 12 kg/mol dimethacrylate-functionalized perfluoropolyether (PFPE-DMA) monomer.

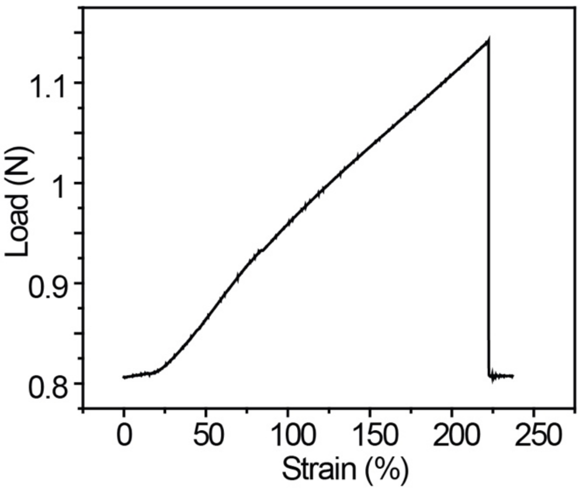

Supplementary Figure 2. Load-strain curve of 12 kg/mol PFPE-DMA film after photocuring.

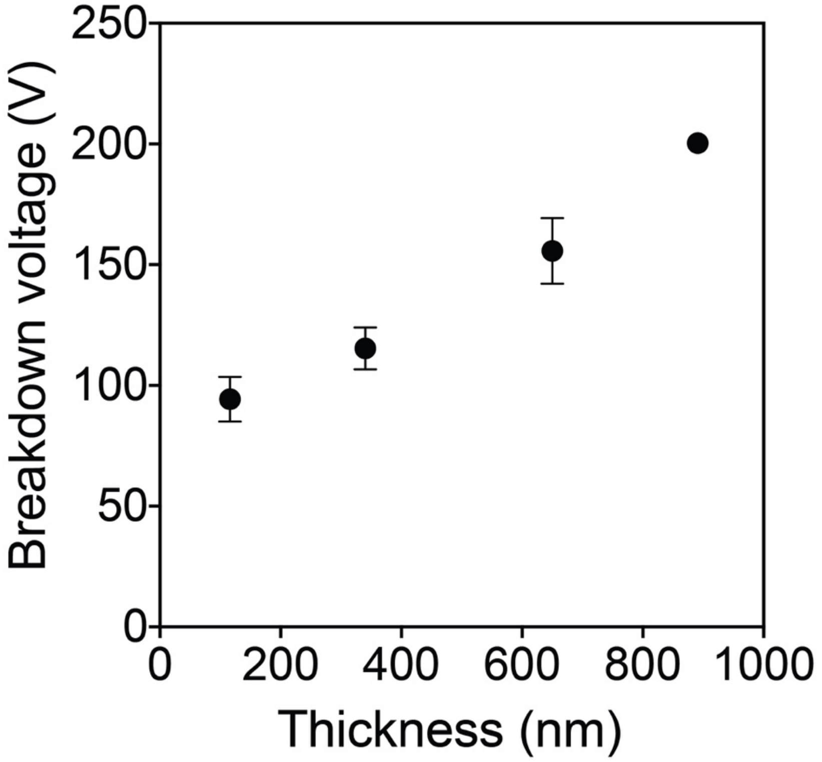

Supplementary Figure 3. Thickness vs. breakdown voltage of spincoated thin film PFPE-DMA. PFPE-DMA was diluted in 1 3-bis(trifluoromethyl)benzene (weight ratio 1:4, 1:3 1:2 and 1:1). 0.3 wt% of 2-Hydroxy-2-methylpropiophenone was added as a photo initiator. The mixed solution was filtered by 0.45 μm filter. Diluted PFPE-DMA was spin coated on the cleaned silicon wafer (as the bottom electrode) at 2000 r.p.m. for 60 s. The corresponding thin film thickness were 116 nm, 340 nm, 650 nm and 891 nm. The spincoated PFPE-DMA films were cured by a 15-min UV curing (365nm) and post-baked at 150 ℃ for 40 mins in the glovebox. A single-wall carbon nanotube (SW-CNT, Carbon Solutions, Inc. P2-SWNT #02-A013) methanol solution (12 mg/70 mL in chloroform, sonication for 20 mins and centrifuge for 30 mins) was spray coated as the top electrodes. The breakdown voltage was measured by Keithley 4200 - SCS parameter analyzer under ambient atmosphere. Values are mean ± S.D..

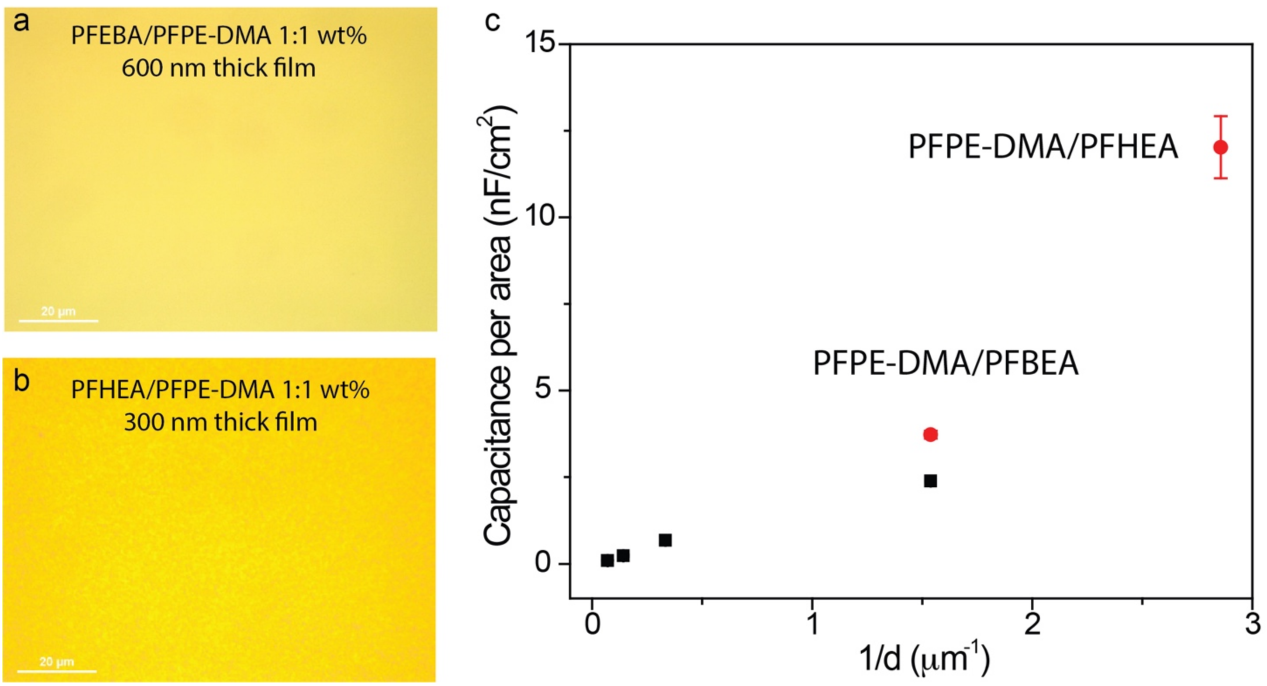

Supplementary Figure 4. Dielectric performance characterization of PFPE-DMA film with different fluorinated acrylate monomer co-polymerization. **a**, optical bright-field (BF) imaging of thin film of co-polymerized (1:1 weight ratio) pentafluorophenyl acrylate (PFBEA) and PFPE-DMA after exposed to solvent listed in Fig. 2. **b**, optical bright-field imaging of thin film of co-polymerized 2-perfluorohexylethyl acrylate (PFHEA) and PFPE-DMA, which show the same level of chemical orthogonality as PFPE-DMA film. **c**, Capacitance per area to width^{-1} of different PFPE-DMA based thin film structure. Black box: PFPE-DMA; red box: PFPE-DMA/PFBEA; red circle: PFPE-DMA/PFHEA. Values are mean ± S.D..

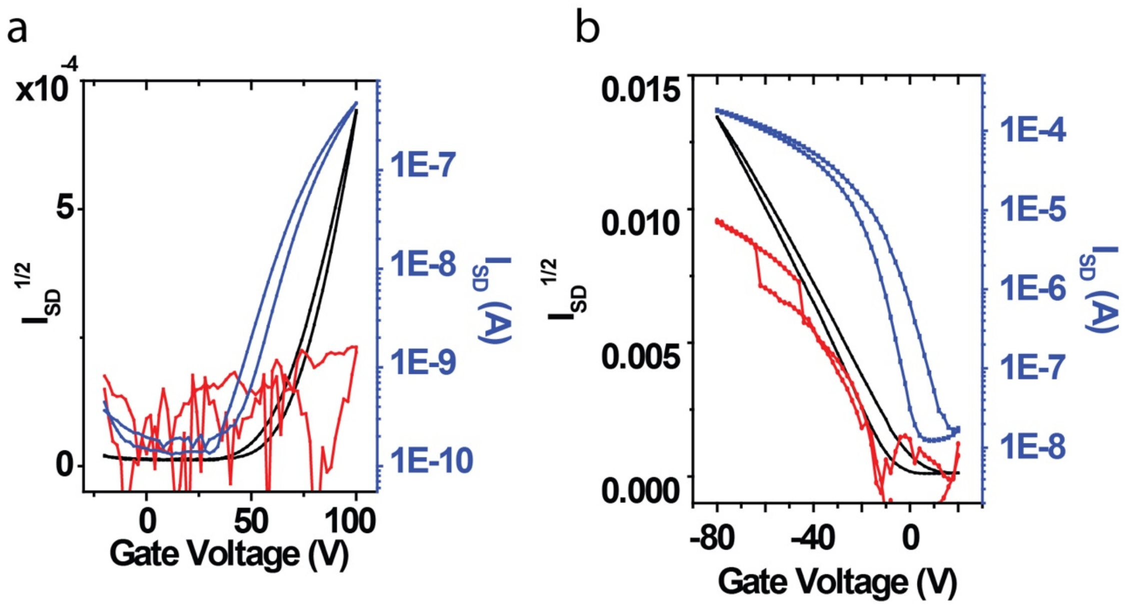

Supplementary Figure 5. Transfer characterization of the polymer FET by using 12 kg/mol PFPE-DMA film as top-gate-bottom-contact structure device. **a**, *n*-type transistor made by semiconductor polymer FBDPPV (Mw = 128.9 kDa, Mn = 66.3 kDa, PDI = 1.94) as previous report[1]. The on current, on/off ratio and mobility are $4.3x10^{-7}$ **a**, $3.2x10^{3}$ and 0.03 $cm^{2}v^{-1}s^{-1}$; **b**, *p*-type transistor made by semiconductor polymer PII2T as discussed above, The on current, on/off ratio and mobility are $1.19x10^{-4}$ A, $5.7x10^{3}$ and 0.40 $cm^{2}v^{-1}s^{-1}$.

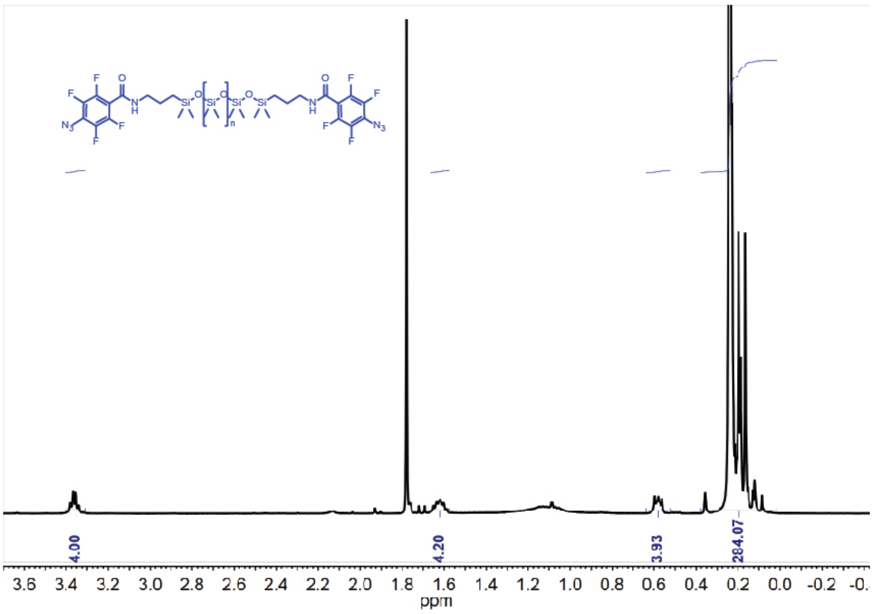

Supplementary Figure 6. ^{1}H NMR of PDMS-azide crosslinker (500 MHz, Chlorobenzene-d_5) δ 3.36 (q, J = 6.8 Hz, 4H), 1.66–1.54 (m, 4H), 0.68–0.49 (m, 4H), 0.31–0.06 (m, 282H).

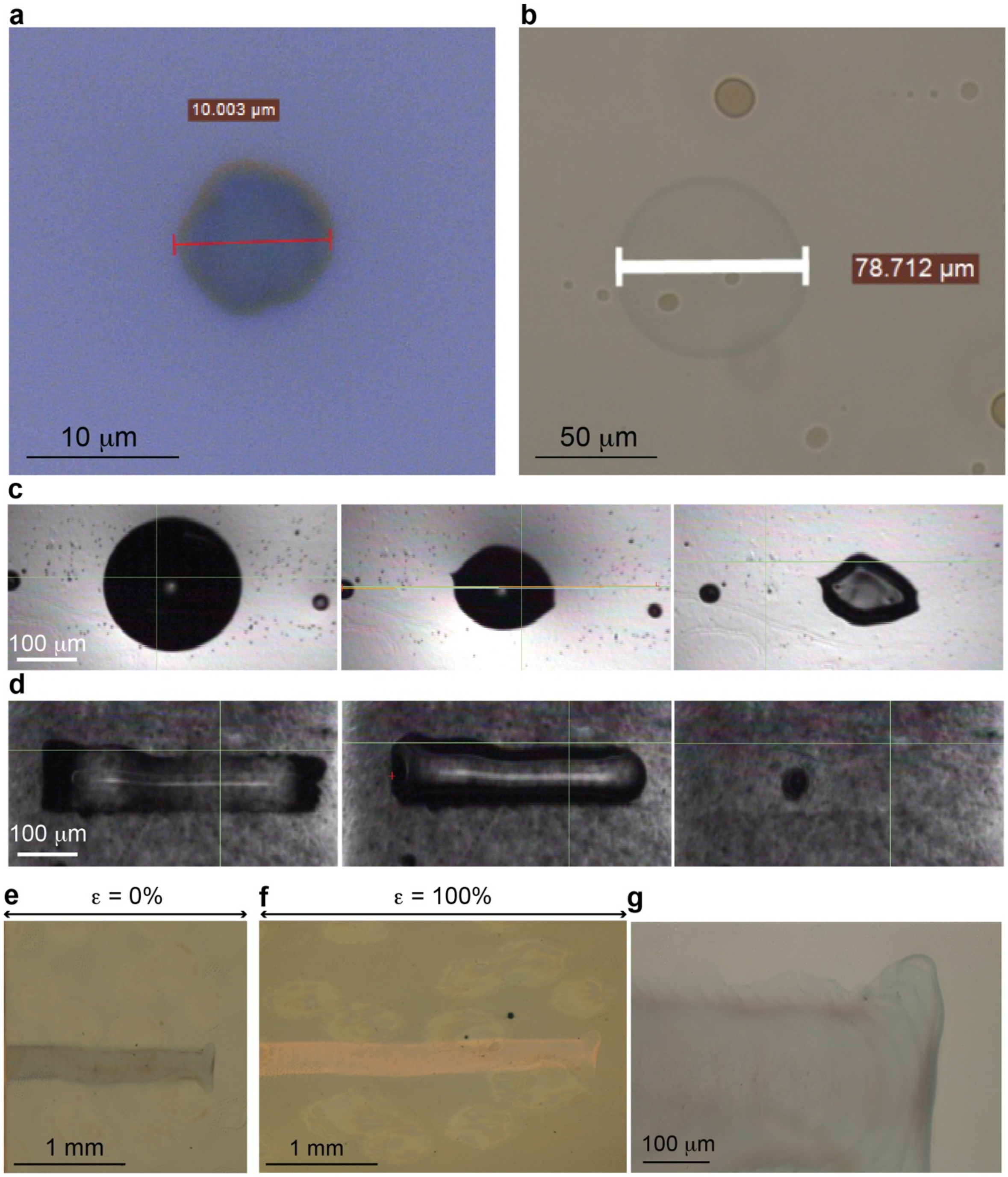

Supplementary Figure 7. Inkjet printing of stretchable PII2T ink (PII2T:PDMS-azide weight ratio 1:1) on PFPE-DMA. **a**, optical BF image of single droplet inkjet printed on PFPE-DMA. **b**, optical BF image of single droplet inkjet printed on the azide crosslinker modified PFPE-DMA. **c-d**, optical BF image of printed PII2T inks on pristine PFPE-DMA substrate (**c**) and modified PFPE-DMA substrate (**d**). **e-f**, optical BF image of a printed line structure on the PFPE-DMA after the thermal annealing at 0% (**e**) and 100% (**f**) strain. **g**, zoomed-in optical BF image of the line structure showing no cracks after 100 cycles of 100% strain.

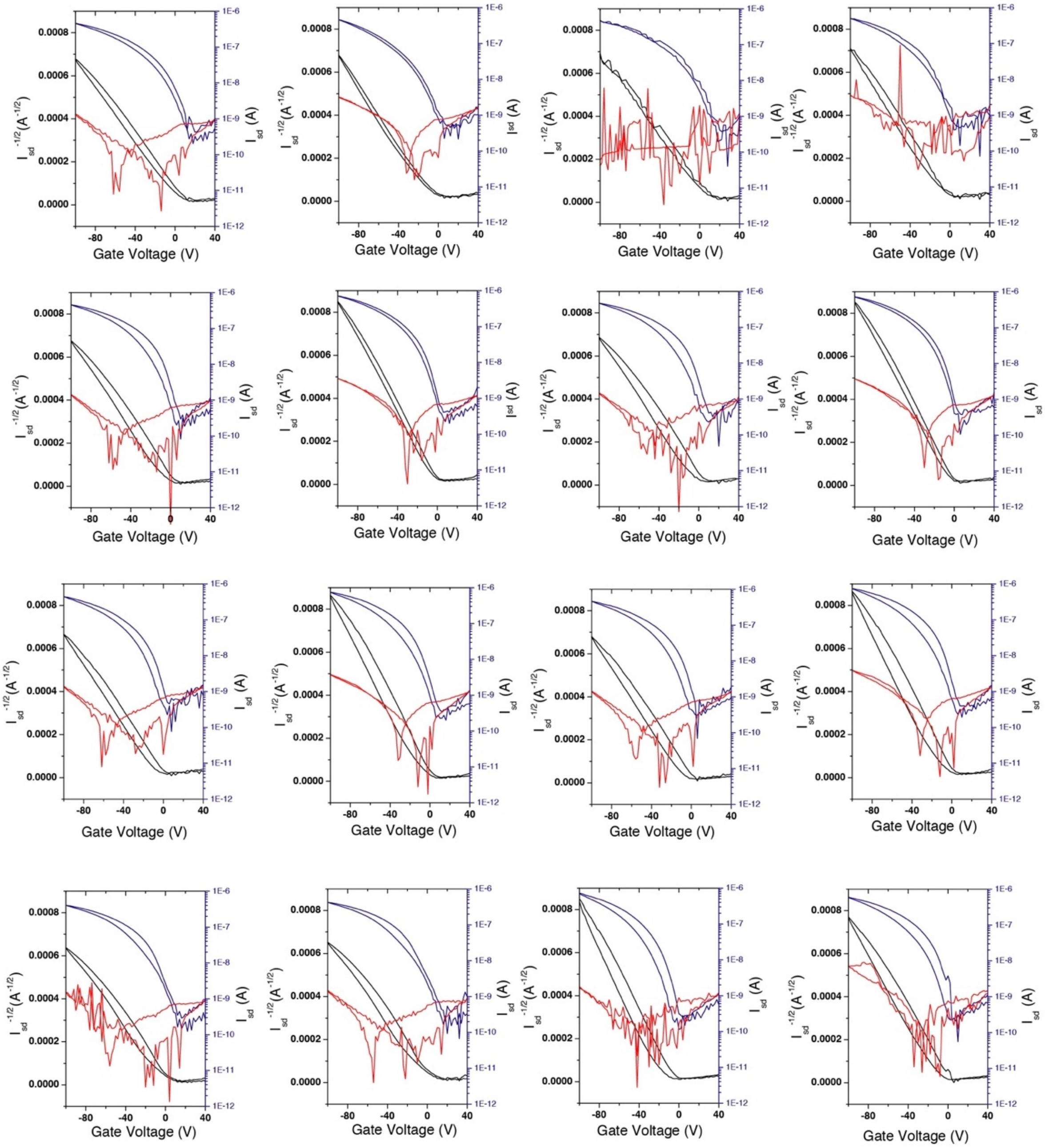

Supplementary Figure 8. Individual transfer curves of 4 x 4 fully stretchable OTFT arrays (V_{sd}=-100V).

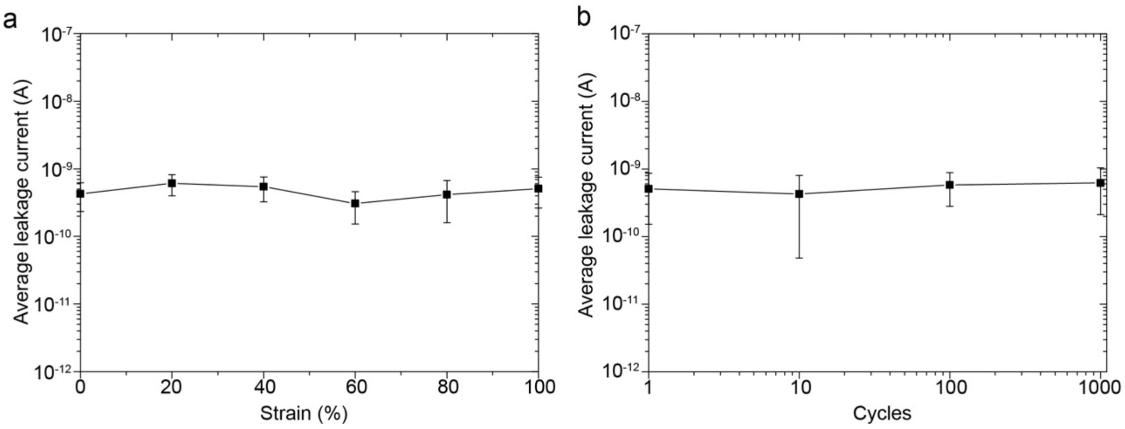

Supplementary Figure 9. Changes in leakage currents with strains up to 100% (**a**) and after multiple stretching-releasing cycles (up to 1000 cycles) at 100% strain (**b**). Values are mean ± S.D..

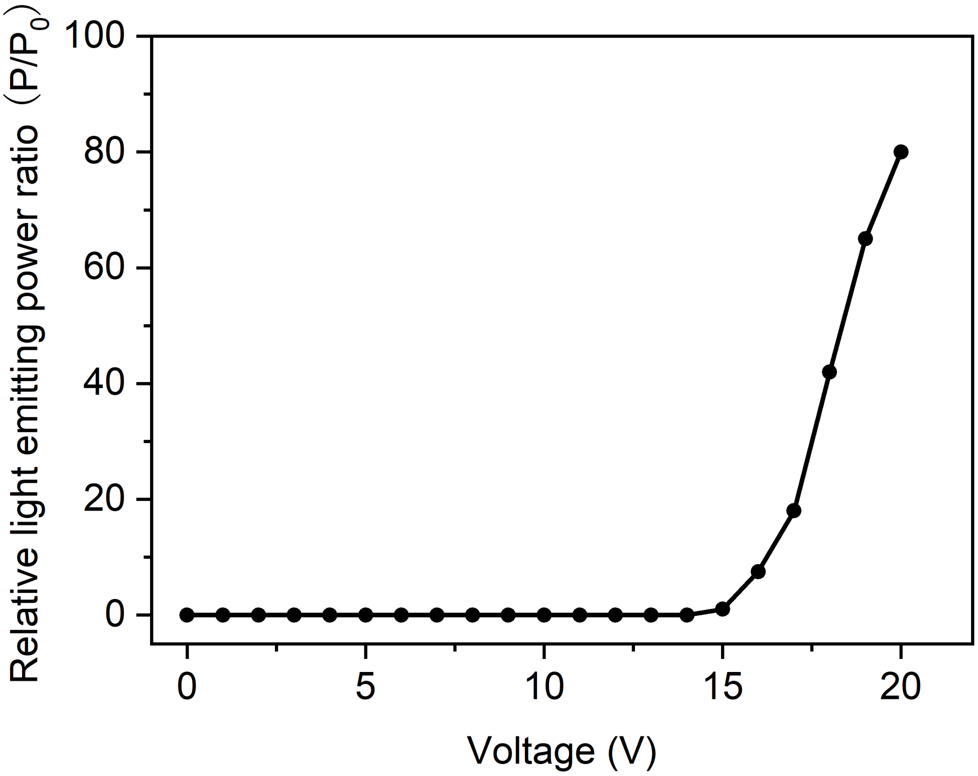

Supplementary Figure 10. The power ratio variation of the OLEC is increased with the increase of applied voltage. The fabrication process of the OLEC was described in the Method section in the main text. For the measurement, we used a power meter (P100D, ThorLabs) to test the brightness change of the OLEC with gradually increasing the applied voltage. P_0 represents the power when the LEC was turned on, and P corresponds the power value of LEC with increasing the applied voltage.

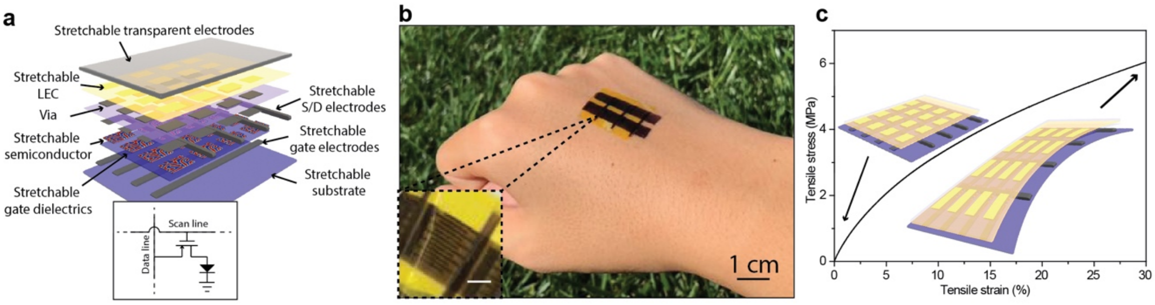

Supplementary Figure 11. Fully stretchable active-matrix organic light-emitting electrochemical cell (AMOLEC) array. **a**, Schematic illustration of the 3D layout of individual components in intrinsically and fully stretchable AMOLEC array. The inset shows the circuit diagram of one single pixel. **b**, Photographic images illustration of an intrinsically stretchable AMOLEC array, which is comprised of 2 x 3 pixels, on a human hand. The inset shows the photographic image of one single pixel from the AMOLEC array. **c**, Strain-stress curve of one AMOLEC array shows 41.7 MPa modulus with 30% stretchability (inset schematics).

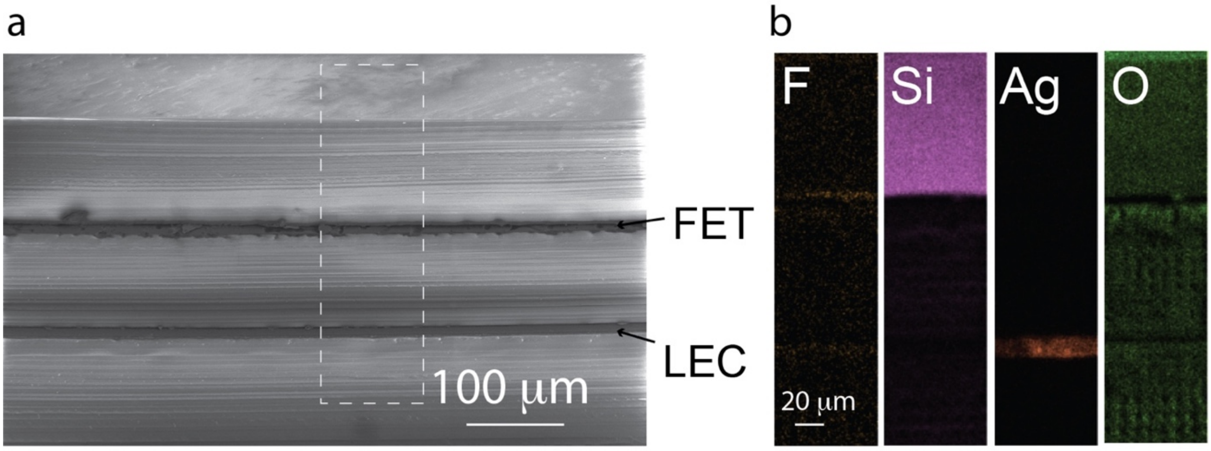

Supplementary Figure 12. SEM and energy-dispersive X-ray (EDX) show vertically integrated and multilayered structure of an AMOLEC skin display pixel. **a**, Cross section of SEM images of an integrated pixel. **b**, EDX element imaging of F, Si, Ag and O elements at the selected regions in (**a**) highlighted by white dashed box.

Supplementary Table

Types of LEC devices	Turn-on voltage (V)	Peak of current density (mA/cm^2)	Stretchability (%)	Reference
**Stretchable**	**6.8**	**35**	**120**	**1**
**Stretchable**	**7**	**70**	**130**	**2**
**Stretchable**	**5**	**12**	**45**	**3**
**Non-stretchable**	**5.6**	**70**	**0**	**4**
**Our stretchable OLEC**	**15**	**7.5**	**30**	**This work**

Supplementary Table 1. Summary of the state-of-the-art stretchable OLEC devices

Supplementary References

1. Liang, J. *et al.*, "Elastomeric polymer light-emitting devices and displays" *Nat. Photonics* **7**, 817 (2013).
2. Liang, J. *et al.*, "Silver nanowire percolation network soldered with graphene oxide at room temperature and its application for fully stretchable polymer light-emitting diodes" *ACS Nano* **8**, 1590 (2014).
3. Yu, Z. *et al.*, "Intrinsically stretchable polymer light-emitting devices using carbon nanotube-polymer composite electrodes" *Adv. Mater.* **23**, 3989 (2011).
4. Zhang, Z. *et al.*, "A colour-tunable, weavable fibre-shaped polymer light-emitting electrochemical cell" *Nat. Photonics* **9**, 233 (2015).
